# Microarray Meta-Analysis Focused on the Response of Genes Involved in Redox Homeostasis to Diverse Abiotic Stresses in Rice

**DOI:** 10.3389/fpls.2015.01260

**Published:** 2016-01-12

**Authors:** Joao B. de Abreu Neto, Michael Frei

**Affiliations:** Abiotic Stress Tolerance, Department of Plant Nutrition, Instituts für Nutzpflanzenwissenschaften und Ressourcenschutz, Rheinische Friedrich-Wilhelms-Universität BonnBonn, Germany

**Keywords:** microarray, meta-analysis, abiotic stress, rice, redox

## Abstract

Plants are exposed to a wide range of abiotic stresses (AS), which often occur in combination. Because physiological investigations typically focus on one stress, our understanding of unspecific stress responses remains limited. The plant redox homeostasis, i.e., the production and removal of reactive oxygen species (ROS), may be involved in many environmental stress conditions. Therefore, this study intended to identify genes, which are activated in diverse AS, focusing on ROS-related pathways. We conducted a meta-analysis (MA) of microarray experiments, focusing on rice. Transcriptome data were mined from public databases and fellow researchers, which represented 36 different experiments and investigated diverse AS, including ozone stress, drought, heat, cold, salinity, and mineral deficiencies/toxicities. To overcome the inherent artifacts of different MA methods, data were processed using Fisher, rOP, REM, and product of rank (GeneSelector), and genes identified by most approaches were considered as shared differentially expressed genes (DEGs). Two MA strategies were adopted: first, datasets were separated into shoot, root, and seedling experiments, and these tissues were analyzed separately to identify shared DEGs. Second, shoot and seedling experiments were classed into oxidative stress (OS), i.e., ozone and hydrogen peroxide treatments directly producing ROS in plant tissue, and other AS, in which ROS production is indirect. In all tissues and stress conditions, genes *a priori* considered as ROS-related were overrepresented among the DEGs, as they represented 4% of all expressed genes but 7–10% of the DEGs. The combined MA approach was substantially more conservative than individual MA methods and identified 1001 shared DEGs in shoots, 837 shared DEGs in root, and 1172 shared DEGs in seedlings. Within the OS and AS groups, 990 and 1727 shared DEGs were identified, respectively. In total, 311 genes were shared between OS and AS, including many regulatory genes. Combined co-expression analysis identified among those a cluster of 42 genes, many involved in the photosynthetic apparatus and responsive to drought, iron deficiency, arsenic toxicity, and ozone. Our data demonstrate the importance of redox homeostasis in plant stress responses and the power of MA to identify candidate genes underlying unspecific signaling pathways.

## Introduction

While most animals can move and escape from harmful conditions, plants cannot. Drought, flood, salinity, extremes of temperature, nutrient deficiency, UV radiation, pollutants, herbicides, and pathogens are some of the factors a plant needs to cope with to survive and grow, usually with more than one at time. These factors limit crop yields and quality (Gill and Tuteja, 2010; Miller et al., 2010; Wang and Frei, 2011). Understanding, how a plant responds to such stresses and the mechanisms underlying stress tolerance can give us a better view of how to improve the global food production. Investigating the stress responses of rice (*Oryza sativa* L.) is particularly rewarding, because it is both a global staple food of great agronomical importance, and a well-studied model organism, for which many transcriptome profiling studies have been published. About 32% of the rice annual production fluctuations (corresponding to around three million tons) can be attributed abiotic stresses (AS), and specifically variations in climate including precipitation and temperature (Ray et al., 2015).

A common responses to different environmental stresses, both abiotic and biotic, is the excessive generation of reactive oxygen species (ROS) including superoxide ($O_{2}^{\cdot -}$), perhydroxy radical (${\mathrm{HO}}_{2}^{\cdot -}$), hydrogen peroxide (H_2_O_2_), hydroxyl radical (OH^⋅^), singlet oxygen (^1^O_2_), and organic hydroperoxide (ROOH; Gill and Tuteja, 2010; Bhattacharjee, 2012). Plant cells continuously produce ROS as by-products of various metabolic processes including respiration and photosynthesis. However, these molecules can directly react with DNA, proteins and lipids causing severe damage to individual cells and whole organisms. For this reason, every aerobic organism tightly controls its ROS concentration by ROS-scavenging pathways composed of many enzymatic and non-enzymatic antioxidant components (Gill and Tuteja, 2010).

However, many studies have shown that ROS play important roles in plants’ stress signaling processes (Fujita et al., 2006; Miller et al., 2010; Mittler et al., 2011; Kim et al., 2012; Pucciariello et al., 2012). There are many advantages in the use of ROS as signaling molecules. The cell can rapidly produce and scavenge different forms of ROS in a simultaneous manner, enabling rapid and dynamic changes in ROS levels (caused by simply tilting the balance between cellular production and scavenging rates; Bhattacharjee, 2012). Each form of ROS has its own characteristics, such as mobility, process and location of origin and its reactivity with DNA, proteins or lipids (Bhattacharjee, 2012). Another advantage is the tight control over the subcellular localization of ROS signals in cells, with the regulation of enzymes specific of each of the cell compartments (Mittler et al., 2011). ROS may even act as “secondary messengers” modulating the activities of specific proteins or expression of genes by changing the redox balance of the cell. The network of redox signals orchestrates the metabolism for regulating energy production to utilization, interfering with primary signaling agents (hormones) to respond to changing environmental conditions at every stage of plant development (Bhattacharjee, 2012). Oxidative signaling is now considered to be a key in the responses to stress, involved not only in the defense to these stresses but also in the regulation of the plant growth and development (Noctor et al., 2014).

One limitation in the understanding of ROS homeostasis in AS is that most of the knowledge on molecular mechanisms of stress response was obtained from experiments under controlled laboratory conditions and focused on only one stress at a time. However, plants are often simultaneously exposed to many biotic and AS in their natural or agronomic habitats (Hazen et al., 2003; Rasmussen et al., 2013). By combining the data from different experiments it is possible to identify common and specific elements expressed in response to different stresses (Rabbani et al., 2003; Fujita et al., 2006; Yang et al., 2013).

With the rapid advances in biological high-throughput technology, a large and diverse set of genomic data has become publicly accessible. Combining information from multiple existing studies can increase the reliability and generalizability of results. The use of statistical techniques to combine results from independent but related studies is called “meta-analysis (MA).” Through MA, we can increase the statistical power to obtain a more precise estimate of gene expression differentials (Ramasamy et al., 2008; Tseng et al., 2012). The most common types of Microarray MA are a combination of *p*-values, a combination of effect sizes (fold change) or a combination of ranks, each method with its limitations and advantages (Tseng et al., 2012).

In the present study, instead of choosing one of those, we used an integrative approach to combine the forces of those different microarray MA methods and overcome possible biases, such as a “fishing for significance” effect for preferring one method over the others (Ioannidis, 2005; Ostlund and Sonnhammer, 2014). However, within these three categories of MA, there are many statistical methods to choose from, we selected them based on their evaluation in recent studies (Tseng et al., 2012; Chang et al., 2013; Ostlund and Sonnhammer, 2014). Among the methods of combination of *p*-values, we chose two distinct methods: Fisher’s (Rhodes et al., 2002), the most commonly implemented MA method, and rOP (*r*th ordered *p*-value) that is more restrictive but still flexible (Song and Tseng, 2014). Between the combined effect size methods, the random effect model, REM is the most adequate for a heterogeneous group of samples, as the one processed in the present study (Choi et al., 2003). Furthermore, the MA by ranks was computed by the program GeneSelector that produces a rank combining seven distinct statistic methods (Boulesteix and Slawski, 2009; Ostlund and Sonnhammer, 2014). Only genes elected by the majority of methods (at least three of those four) were further processed in our analyses. Although this strategy may be rather restrictive, it increases the power of our analysis, since each differentially expressed gene (DEG) was confirmed by at least three different statistic methods.

The goal of this MA was to detect DEGs involved in ROS homeostasis, which respond to AS treatments. The transcriptome data from rice plants subjected to different classes of AS were analyzed: drought, submergence, salinity, cold, heat, excess and/or deficiency of essential nutrients, such as phosphorus (P), zinc (Zn), and iron (Fe), and toxicity of heavy metals such as arsenic (As), cadmium (Cd), chrome (Cr), and lead (Pb). Also, to identify genes specifically involved in the redox homeostasis, data from experiments with ozone (O_3_) and hydrogen peroxide were included (**Table 1**), because these treatments are presumed to produce direct oxidative stimulus, unlike with the indirect forms of oxidative stress (OS) occurring in other environmental conditions.

**Table 1:** Experimental conditions of microarray raw data used for meta-analysis.

Experiments			Source		
Shoots	Roots	Seedlings	GEO series	Reference	Platform
O_3_			GSE11157	Cho et al., 2008	GPL892
O_3_			NA	Frei et al., 2010a	GPL892
Submergence			GSE18930	Mustroph et al., 2010	GPL2025
Drought			GSE21651	Pandit et al., 2011	GPL2025
Salinity			GSE21651	Pandit et al., 2011	GPL2025
–N			GSE66935	Takehisa et al., 2015	GPL6864
–K			GSE66935	Takehisa et al., 2015	GPL6864
–P			GSE66935	Takehisa et al., 2015	GPL6864
–P			GSE17245	Zheng et al., 2009	GPL2025
–Fe			GSE17245	Zheng et al., 2009	GPL2025
–Fe –P			GSE17245	Zheng et al., 2009	GPL2025
–P	–P		GSE6187	Pariasca-Tanaka et al., 2009	GPL892
+Fe	+Fe		NA	Wu et al., unpublished data	GPL19782
–Zn	–Zn		NA	Wu et al., unpublished data	GPL19782
–Zn +Fe	–Zn +Fe		NA	Wu et al., unpublished data	GPL19782
Drought	Drought		GSE26280	Wang et al., 2011	GPL2025
	+As		GSE25206	Dubey et al., 2010	GPL2025
	+Cd		GSE25206	Dubey et al., 2010	GPL2025
	+Cr		GSE25206	Dubey et al., 2010	GPL2025
	+Pb		GSE25206	Dubey et al., 2010	GPL2025
	–K		GSE37161	Ma et al., 2012	GPL2025
	Salinity		GSE14403	Cotsaftis et al., 2011	GPL2025
		H_2_O_2_	GSE19983	Mittal et al., 2012a	GPL9956
		Cold	GSE19983	Mittal et al., 2012a	GPL9956
		Heat	GSE19983	Mittal et al., 2012a	GPL9956
		H_2_O_2_	GSE32704	Mittal et al., 2012b	GPL8852
		H_2_O_2_ + cold	GSE32704	Mittal et al., 2012b	GPL8852
		H_2_O_2_ + heat	GSE32704	Mittal et al., 2012b	GPL8852
		heat	GSE14275	Hu et al., 2009	GPL2025
		Cold	GSE6901	Jain et al., 2007	GPL2025
		Drought	GSE6901	Jain et al., 2007	GPL2025
		Salinity	GSE6901	Jain et al., 2007	GPL2025
		Salinity	GSE16108	Pandit et al., 2011	GPL2025
		+As	GSE4471	Norton et al., 2014	GPL2025
		–K	GSE44250	Shankar et al., 2013	GPL2025
		–Pi	GSE35984	Dai et al., 2012	GPL2025

More specifically, the following questions were addressed in this study:

(1) How does a MA integrating several of the MA approaches described above compare to individual MA approaches, and are the results more conservative?
(2) To what extent do stress responses differ between different stresses and plant tissues, and what is the role of ROS-related genes?
(3) Through MA, can we nominate possible key genes as major hubs of ROS-related stress signaling, and what are their putative functions?

## Materials and Methods

### Data Mining

For this study, expression data of rice plants exposed to diverse AS were combined. The raw expression data of different experiments were obtained from the Rice Oligonucleotide Array Database^1^ (Cao et al., 2012), the NCBI’s Gene Expression Omnibus repository^2^ (Barrett et al., 2013), and from fellow researches. The criteria for inclusion of those dataset were: relatively similar genetic background, plants must be from *O. sativa* indica or japonica subgroups; the RNA should have been extracted only from shoots, roots, or seedlings (whole plant), excluding other tissues such as flowers, seeds, or *cali*; the experiments must involve only against AS treatments; the data must originate from Affymetrix or Agilent microarray platforms, and the original study must follow the “Minimal Information About a Microarray Experiment” (MIAME) requirements (Brazma et al., 2001).

### Individual Datasets Analysis

Microarray expression data from each source study was pre-processed separately as individual datasets. Agilent microarray data were processed in the R program using the packages LIMMA (Smyth, 2005), while the package Affy was used for data from the Affymetrix platforms. The raw data from both were treated using Robust Multi-array Average (RMA) background correction and quantile normalization. Non-informative and low-intensity probes were declared following the program standard settings, while duplicated probes were converted into their corresponding genomic locus. The ArrayQualityMetrics package (Kauffmann and Huber, 2013) was used to assess the quality of the normalized datasets. A sample was included or excluded in further analysis based on three different evaluations made by this program: distance between array, array intensity distributions, and individual array quality. The values normalized by RMA were used for the subsequent MA.

A contrast between each treatment and its control was estimated with the LIMMA package. In studies including several genotypes the genotype factor was not considered. After fitting the data into a linear model, the standard errors were corrected using a simple empirical Bayes model. Moderated *t*-statistic and log-odds of differential expression were computed for each contrast for each gene. Genes that showed significant *P*-value (*FDR* = 5%) were considered as DEG and log_2_-fold-change values of each experiment/dataset were saved for further analysis. Relative gene expression values corresponding to the same stress in different datasets were averaged for a simplified evaluation of the gene’s response to each condition.

### Combined Meta-Analysis

Two different strategies were implemented in the present study. The first (MA.1) investigated the effect of the stresses in different tissues, for which the datasets were separated into shoots, roots and seedling microarrays and processed separately. In the second approach (MA.2), data from shoots and seedlings were combined, while the data set was separated into direct OS and others AS to compare the effects of direct and indirect OS on gene expression. Hydrogen peroxide and ozone stress were considered as OS, because these treatments directly lead to the production of ROS in plant tissue (Uchida et al., 2002; Kangasjärvi et al., 2005). For both approaches, the normalized expression values were used. Since the probe nomenclature differs between platforms, the MSU-ID was used, and when multiple probes matched to the same gene they were averaged.

The datasets were merged using the packages metaDE (Wang et al., 2012), and this merged dataset was once more filtered, excluding 20% of un-expressed genes (with small expression intensities) and 20% of non-informative genes (genes with small variation). Each independent study sample was processed with a modified two-sample *t*-statistics (modt) contrasting treated and control samples.

In an effort to overcome the inherent artifacts of each MA statistical method, we ran our data through the three common types of MA (i.e., by *p*-value, by effect size and by rank) to identify genes that are considered differentially expressed by distinct methods. Two methods of combination by *p*-value were used: Fisher and rOP. The classical Fisher’s method sums the log-transformed *p*-values obtained from individual studies and, under null hypothesis, follows a chi-squared distribution with 2K degrees of freedom, where K is the number of studies combined (Rhodes et al., 2002). However an extremely small *p*-value in only one study can be sufficient to cause statistical significance, even if the same gene are not significant in any other study. A more restrictive but flexible method is rOP, that combines Fisher with a generalized vote counting statistic. It uses the *r*th order statistic among sorted *p*-values of K combined studies, where *r* is a pre-determined minimum number of studies, in which a gene’s *p*-values must be small to be significant (Song and Tseng, 2014). In our analysis we implemented K/2 ≤*r* ≤ K, i.e., each gene must be differentially expressed in at least half of the combined studies to be significant. Taking into consideration the heterogeneity of our cluster of studies and the residual “noise” data derived from technical and biological differences between the studies, a more restrictive approach was not implemented (i.e., *r* = K).

A second way to combine expression data across different microarray studies and platforms is using effect size values. The REM method was implemented because it possesses a random effect element corresponding to the unknown heterogeneities between very distinct studies, such element is not present in the alternative method, the fixed effect model, FEM (Choi et al., 2003). The results of each of these analyses were corrected with the Benjamini and Hochberg procedure with a false discovery rate (FDR) of 5% as threshold.

For a MA based on the rank method, the normalized values were processed using GeneSelector (Boulesteix and Slawski, 2009). This package allows a ranking analysis of the data with seven distinct methods: ordinary *t*-test; Baldi and Long Bayesian *t*-test (Baldi and Long, 2001); Winconxon–Mann–Whitney *U* test; Fox and Dimmic Bayesian *t*-test (Fox and Dimmic, 2006); SAM statistics (Tusher et al., 2001); limma: moderated *t*-statistic based on a Bayesian hierarchical model which is estimated by an empirical Bayes approach (Smyth et al., 2003), and simple fold-change estimation (in log_2_). The obtained gene rankings were then aggregated by the mean value of the rank positions given by each method (AggMean), or aggregated on the basis of a Markov Chain model, AggMC (DeConde et al., 2006). A combined rank was produced by combining the top genes obtained by each of the seven ranking methods, together with the AggMean and AggMC lists. Genes of this combined list were compared with DEGs obtained by Fisher, rOP, and REM methods.

The DEGs shared by at least three of these four methods were further studied.

### Gene Analysis

The genes elected by the combined meta-analyses MA.1 and MA.2, were further analyzed and characterized. First, the result lists were combined and compared with a list of genes described in the literature as involved in ROS scavenging and signaling processes. This list was curated based on recent studies (Frei et al., 2010a; Liu et al., 2010; Kim et al., 2012; Shaik et al., 2014). This list includes genes involved in the biosynthesis and recycling of enzymatic [such as superoxide dismutase (SOD), catalase (CAT), ascorbate peroxidase (APX), glutathione peroxidase (GPX), and glutathione reductase (GR)) and non-enzymatic antioxidants (such as ascorbic acid (AsA), reduced glutathione (GSH) and thioredoxin (Trx)], and also transcription factors such as zinc-fingers, MYB (**my**elo**b**lastosis) and WRKY (that contains the WRKYGQK amino acid conserved sequence) families and other elements described as directly or indirectly in involved in signaling and response to OS. Co-occurrence of DEG with the resulting list was represented with Venn’s diagrams using Venny 2.0 (Oliveiros, 2015).

Gene Ontology (GO) Enrichment analysis of the DEGs obtained by MA.1 and MA.2 was conducted using the AgriGO platform (Du et al., 2010). A Singular Enrichment Analysis was performed using the Rice Gramene Locus set as reference (Jaiswal et al., 2002).

Genes differentially expressed in both OS and AS (MA.2 approach) were studied in depth. The STRING (Search Tool for the Retrieval of Inter-acting Genes/Proteins) database was used to detect functional association between those genes. This database constructs associations based on distinct lines of evidences: Experimental evidence from protein–protein interaction assays; co-expression data based on the expression data stored in the NCBI GEO database; co-occurrence of the genes in the same organisms; available information of other databases; recurring neighborhood of the genes in known genomes; events of fusion between those genes or orthologs; pathway annotation data in other databases such as Gene Ontology or Kyoto Encyclopedia of Genes and Genomes (KEGG), and automated text-mining based on Medline abstracts and a large collection of full-text articles (Szklarczyk et al., 2015). STRING computes a confidence score for those interactions based on the available evidences, from medium (score above 0.4) to highest (above 0.9).

Using the expression values obtained in the individual dataset analysis (a log_2_ fold change difference between control and treated expression values of each gene), an average value of the most relevant conditions was obtained and represented in a heatmap. The heatmap was made using the package gplots in R (Warnes et al., 2015).

## Results

### The Percentage of ROS Related DEGs is Constant in the Response to Different Stresses

After search in the available databases and quality control analysis, raw microarray data from 36 independent experiments were selected. To obtain a global analysis, AS from different categories were chosen: drought, submergence, salinity, cold, heat, excess and/or deficiency of essential nutrients as Phosphorus (P), zinc (Zn), and iron (Fe), heavy metal toxicity (As, Cd, Cr, and Pb), and direct OS (O_3_ and H_2_O_2_; **Table 1**).

Some source data originated from experiments that tested different stressors (Jain et al., 2007; Zheng et al., 2009; Dubey et al., 2010; Mittal et al., 2012a,b); several time points after the exposure to the stressor (Cho et al., 2008; Mustroph et al., 2010; Dai et al., 2012; Ma et al., 2012; Mittal et al., 2012a); several concentrations of a stressor (Takehisa et al., 2015), and/or several genotypes, usually using contrasting lines (Pariasca-Tanaka et al., 2009; Frei et al., 2010a; Cotsaftis et al., 2011; Pandit et al., 2011; Norton et al., 2014; Supplementary Table S1). In these cases, every possible contrast of stress condition versus control was treated as a separate experiment. In total, plants from 21 different genotypes of domestic rice were used, 13 from the *indica* and eight from the *japonica* subspecies (Supplementary Table S1).

In parallel, a list of 1972 genes previously described as involved in ROS scavenging and signaling processes was made by combining information from the literature (Frei et al., 2010a; Liu et al., 2010; Kim et al., 2012; Shaik et al., 2014). These genes correspond to about 4% of the rice genes represented by the microarray probes included in this analysis (Supplementary Table S2) and represent a broad spectrum of functions, ranging from reductase and peroxidase enzymes to transcription factors.

In the different treatments and tissues, 7–10% of the DEGs were included in the list of ROS-related genes, indicating that this category of genes was overrepresented (**Table 2**). The proportion of DEG considered as ROS-related was similar in OS and AS experiments (**Table 3**).

**Table 2:** Differentially expressed genes (DEGs) in response to different abiotic stresses and the proportion of those involved in ROS scavenging or signaling mechanisms (ROS).

Shoots			Roots			Seedlings		
	DEG	ROS		DEG	ROS		DEG	ROS
Drought	17682	7%	Drought	11410	7%	Drought	9756	7%
Salinity	7268	8%	Salinity	3625	8%	Salinity	11242	7%
–P	18116	8%	–P	482	10%	–P	15704	7%
–Fe	13548	8%	+Fe	7938	7%	Cold	11347	8%
–Zn +Fe	13679	7%	–K	4243	7%	Heat	14692	8%
O_3_	11979	8%	+Cd	1011	10%	–K	6275	7%
Submergence	14001	7%	+As	8581	7%	H_2_O_2_	15198	7%
			+Pb	285	11%	+As	7599	8%
TOTAL	23197	7%	TOTAL	21273	7%	TOTAL	21533	7%

*Total number of DEGs detected in at least one dataset in each sample cluster: shoots, roots, and seedlings (FDR = 5%).*

**Table 3:** Detected number of genes differentially expressed in response to abiotic stresses.

Meta-analysis	MA.1			MA.2	
	Shoots	Roots	Seedlings	OS	AS
**(A) Samples**					
Number of studies	9	6	8	4	17
Experiments	32	22	23	10	67
Samples	123	104	87	42	167
DEGs	6336	6657	7988	7370	6966
**(B) Number of differentially regulated genes in each MA approach**					
Fisher	5770 (91%)	3335 (50%)	6860 (86%)	2972 (40%)	6807 (98%)
rOP	5313 (84%)	3582 (54%)	6610 (83%)	2515 (34%)	6668 (95%)
REM	1058 (17%)	1317 (20%)	1199 (15%)	1320 (18%)	1707 (25%)
GeneSelector	100 (2%)	100 (2%)	100 (1%)	100 (1%)	100 (1%)
Shared DEG	1001 (16%)	837 (13%)	1172 (15%)	990 (13%)	1727 (25%)
**(C) ROS related genes differentially expressed in each MA approach**					
Total DEGs	468 (7%)	540 (8%)	502 (6%)	548 (7%)	466 (7%)
Fisher	431 (7%)	339 (10%)	500 (7%)	271 (9%)	466 (7%)
rOP	398 (7%)	349 (10%)	499 (8%)	243 (10%)	465 (7%)
REM	79 (7%)	100 (8%)	119 (10%)	129 (10%)	142 (8%)
GeneSelector	7 (7%)	9 (9%)	4 (4%)	11 (11%)	12 (12%)
Shared DEG	72 (7%)	73 (9%)	116 (10%)	97 (10%)	148 (9%)

***(A)** Number of individual studies, experiments in which the contrast between treatment and control was evaluated, biological samples (microarrays) and number of differentially expressed genes (DEGs) in at least one dataset. **(B)** DEGs obtained in each MA statistical approach (FDR = 5%), and the proportion in relation to the total number of DEGs in parenthesis. Shared DEGs are genes elected by the majority of methods. **(C)** Number and proportion of the ROS-related genes in the DEGs obtained by each approach **(B)**. More details in Supplementary Tables S1–S4.*

### MA.1: DEGs in Response to Diverse Abiotic Stresses Differ Between Tissues

**FIGURE 1:**
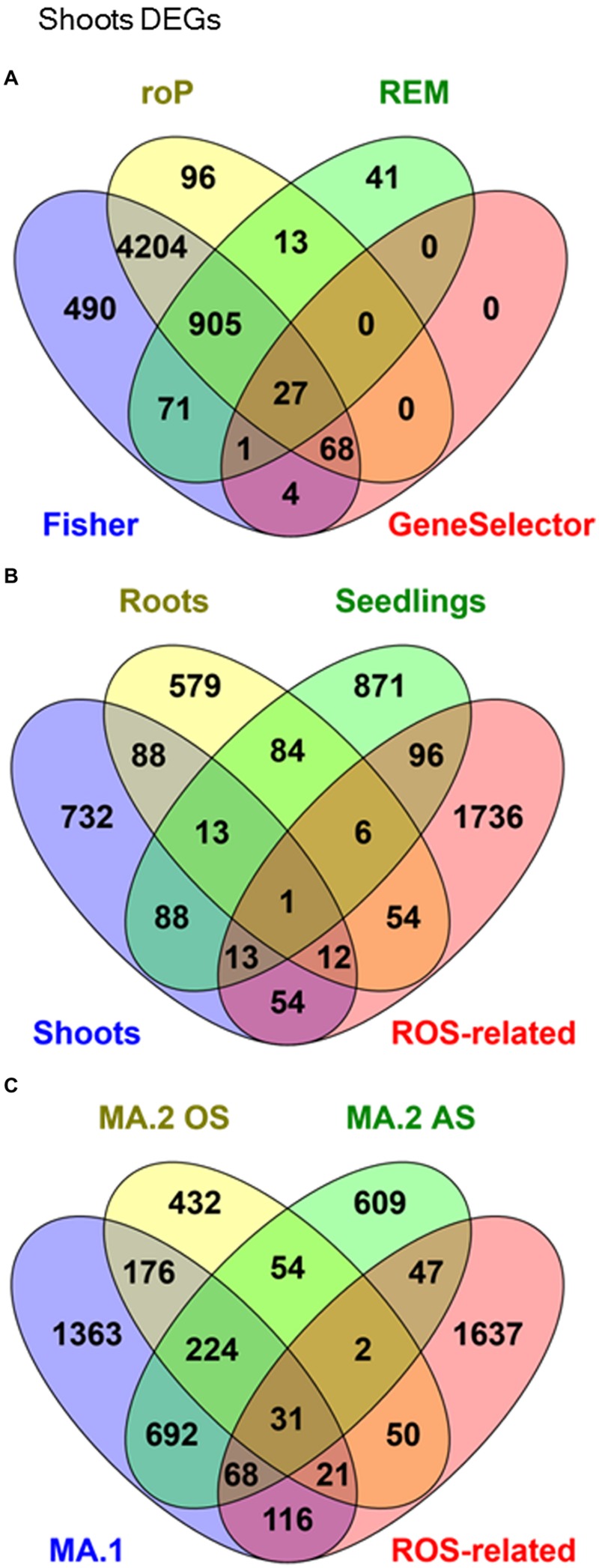
**Venn Diagrams showing co-occurrence of differentially expressed genes (DEGs) in response to many abiotic stresses in: **(A)** Shoots samples processed by four different meta-analytical (MA) statistical methods: Fisher, rOP, REM, and GeneSelector. (B)** MA.1 combined analysis of shoots, seedlings, and roots samples, compared with an a priori ROS-related gene list. **(C)** Oxidative stress (MA.2 OS) and other abiotic stresses (MA.2 AS), compared with MA.1 and ROS-related genes.

The microarray expression data was processed by two different MA approaches. In the first, MA.1, our objective was to identify DEGs in response to many AS in shoot, root, and seedling samples separately. Using combined MA methods, 1001 DEGs were identified in response to different stresses in shoots, 837 in roots and 1172 in seedlings, although only 14 were identified in all three tissues (**Figure 1B**, **Table 3**). From the 2691 genes elected by MA.1, 236 (9%) were included in the ROS-related list, including the coding genes of 42 zinc-finger signaling proteins, 18 MYB transcript factors, nine glutathione S-transferases, a Cu-Zn SOD, a copper chaperone for SOD, 19 peroxidase precursors, APX2, GPX2, and GPX3 (**Figure 1B**, Supplementary Table S5).

### MA.2: DEGs Detected in Both Oxidative and Others Abiotic Stresses

In a second approach, expression data from seedling and shoot experiments were classed into two groups: OS (direct OS, i.e., O_3_ and H_2_O_2_) and AS (all the other AS). It was found that 679 DEGs were exclusive to OS and 1416 DEGs were exclusive to AS (**Table 3**, **Figure 1C**). In total 1212 genes were detected by both MA.2 and MA.1, of which 197 coincided with OS, 760 with AS, and 255 with both (**Figure 1C**). On the other hand, 50 ROS-related genes were only detected by the OS analysis, including APX7 and nine WRKY proteins, and 47 by the AS analysis (Supplementary Table S5).

We then focused on the 311 DEGs shared by both OS and AS. Among those, 33 were ROS-related genes, while others belonged to many distinct gene families associated with the response to stress and plant growth, such as Zn fingers, WRKY, and TIFY transcript factors, cytochromes, photosystem subunits, heat shock proteins, HIPPs (Heavy Metal Isoprenylated Plant Proteins), kinases and phosphatases (Supplementary Table S5).

**FIGURE 2:**
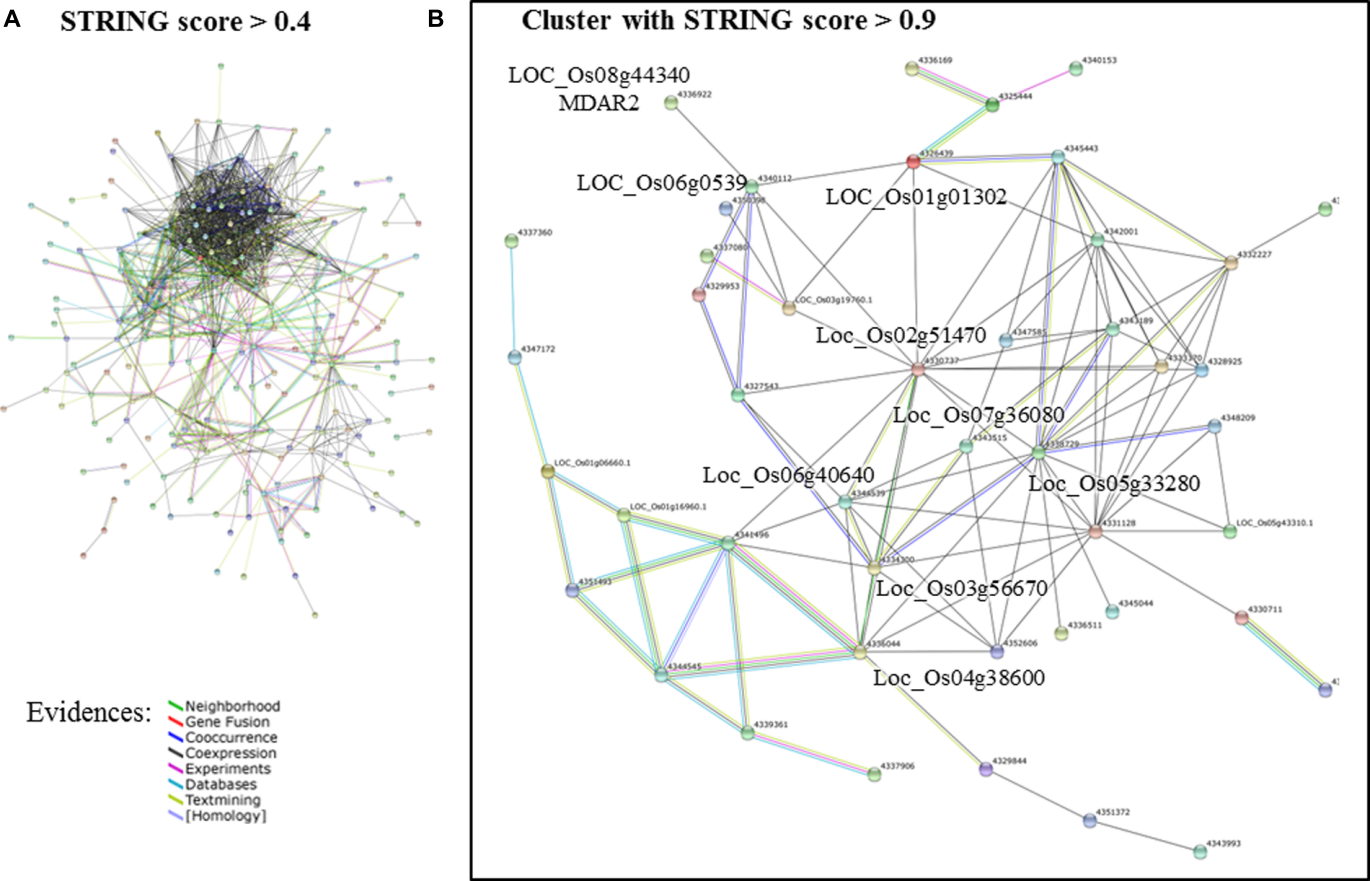
**Network analysis of the DEGs identified both in oxidative (OS) and abiotic stress (AS) analyses of MA.2 approach. (A)** Networks formed by STRING 10.0 database shows all connections of those genes with a confidence score > 0.4. **(B)** Even with a more conservative analysis (confidence score > 0.9), 47 of those genes formed a cluster (genes listed in **Figure 3** and Supplementary Table S5). The connection colors represent the types of evidence for inferring association: recurring Neighborhood in different genomes (green line), events of Gene Fusion (red), Co-occurrence of those genes in the same organisms (dark blue), co-expression (black), Experimental protein–protein interaction data (pink), pathway described by other databases (light blue), literature text-mining (yellow), and homology (purple lines). Source: STRING 10.0 (Szklarczyk et al., 2015).

**FIGURE 3:**
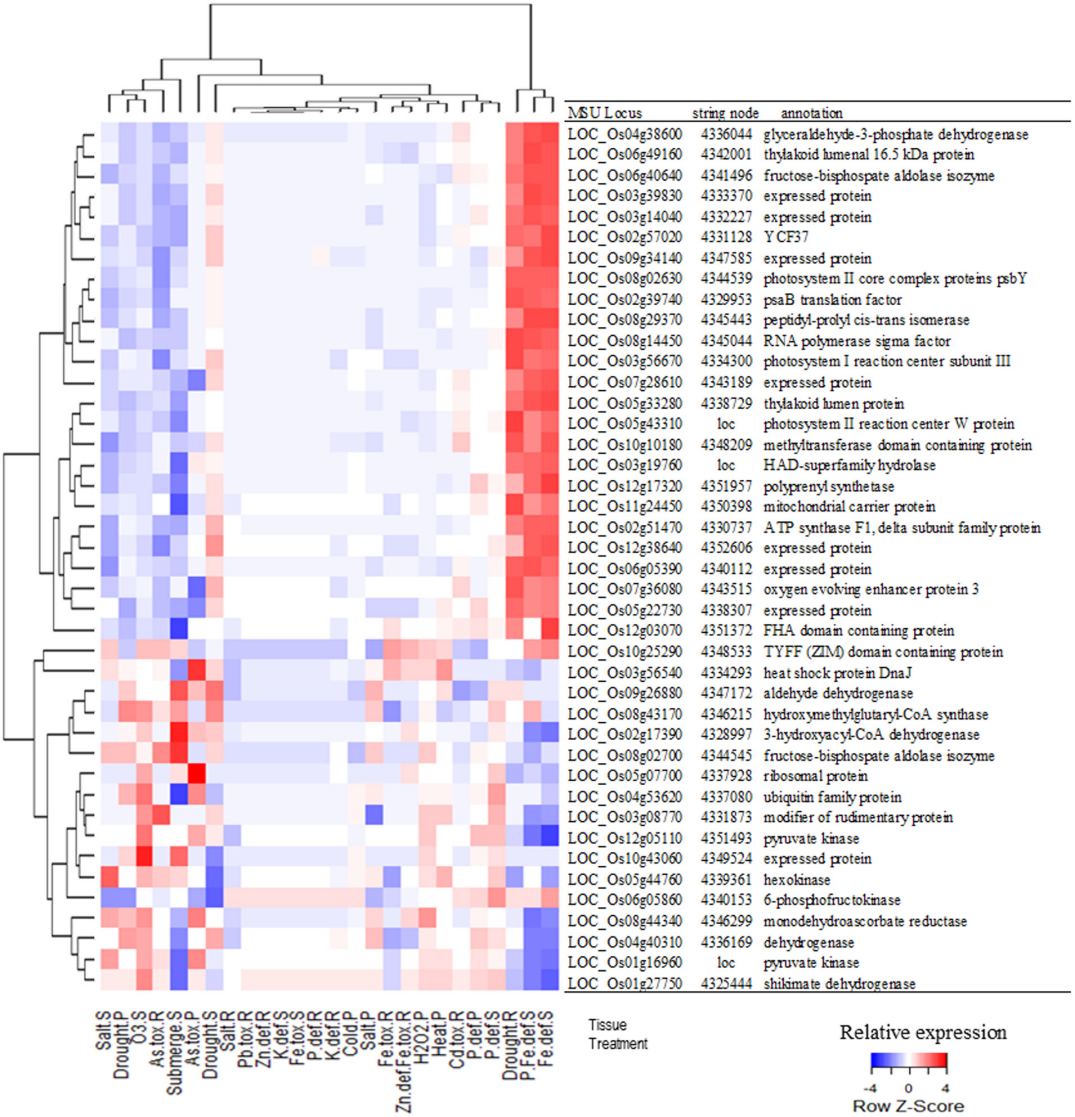
**Relative expression of the genes shown in **Figure 2B** in response to different treatments and in different tissues (R, Roots; S, Shoots; and P, Seedlings) followed by each gene’s MSU locus, STRING node id and description.** Each gene is represented in **Figure 2B** as a node identified by a STRING node number or the locus name (loc). Color scale shows relative expression of each gene in relation to all tested treatments.

A network analysis using the platform STRING 10.0 detected a cluster between 214 of those genes (**Figure 2A**). By increasing the stringency of this analysis (confidence score > 0.9), it was possible to isolate a cluster of 47 protein-coding genes (**Figure 2B**). Most hub genes of this network code proteins involved in the photosynthetic apparatus, e.g., ATP synthase (LOC_Os02g51470), oxygen evolving enhancer protein 3 (LOC_Os07g36080), photosystem I reaction center subunit III (LOC_Os03g56670), photosystem II core complex proteins psbY (LOC_Os08g02630), photosystem II reaction center W protein (LOC_Os05g43310) and many others chloroplastic protein (Supplementary Table S5). Many of these genes showed a similar expression pattern in the samples analyzed in this study (Supplementary Table S7). They were more highly expressed in response to iron deficiency and drought in roots, while suppressed in the samples of submitted to As toxic level, ozone, and submergence (**Figure 3**).

### Shared DEGs are Involved Many Metabolic, Response to Stimuli, and Regulatory Processes

**FIGURE 4:**
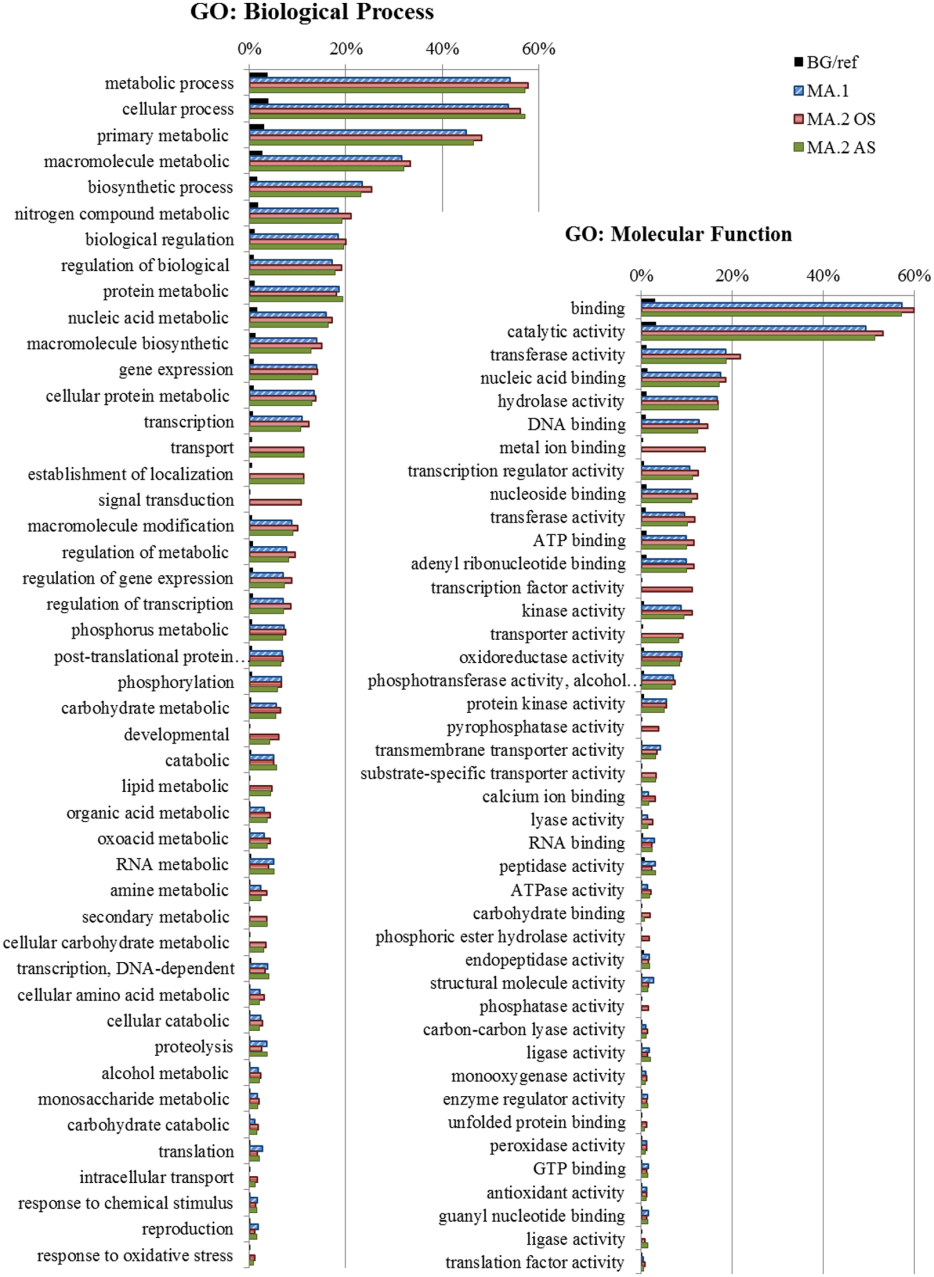
**Gene Ontology (GO) – Frequency of the most representative Biological Process terms.** In blue DEGs obtained by the first meta-analysis approach (MA.1), in red by oxidative stress (MA.2 OS) and in green by abiotic stress (MA.2 AS) of the second approach. The frequency of these terms in the reference background (BG), the Rice Gramene Locus set is shown in black. GO analysis made in the AgriGO platform (FDR = 5%).

Gene Ontology enrichment analysis was conducted to explore other possible functions of the DEGs detected in the different MA approaches (Supplementary Table S6). The most frequent and significant GO terms associated with DEGs in MA.1, MA.2, AS, and OS are represented in **Figure 4**. The biological processes terms suggest constitutive roles for those genes, as part of metabolic and biosynthetic processes, but also in the regulation of those processes on different levels (transcription, post-translational protein modification, macromolecule biosynthesis, phosphorylation, signal transduction, transport, and proteolysis). The enrichment of terms such as nucleic acid binding, transcription regulator, kinase activity, transmembrane transporter, and phosphatase activity indicates that many of those shared genes are also involved in signaling processes (**Figure 4**).

## Discussion

### Advantages of a Combined MA Approach

With the objective of identifying genes involved in the plant response to different AS and direct or indirect OS, the expression data of rice plants exposed to many stress conditions were combined. Instead of using one specific MA approach, we combined the results of different MA statistical methods to obtain the most relevant genes. The stringency of this methodology may exclude many important genes, but also excludes many false positives that could be obtained as artifacts of each statistical method (Tseng et al., 2012; Chang et al., 2013).

While methods such as Fisher’s identify a great number of genes, many of those were not identified as differentially expressed by size effect or ranking product methods (e.g., **Figure 1A**). Only a small fraction of the genes differentially expressed was shared between the independent methods. For example, while with the Fisher’s MA method the number of DEGs was between 40 and 98% of the number of DEGs detected in at least one experiment, our shared DEGs approach reached 13–25% (**Table 3B**).

### MA.1: The Response to Stresses Varies Between Tissues

In our first MA approach, microarray data were separated into shoots, roots, and seedlings, and although those groups were composed of samples of similar size (**Tables 1** and **2**), they showed greatly distinct numbers of DEGs in response to stresses, and only 14 of those were shared between the three groups (**Figure 2B**). This is in agreement with the concept that the transcriptome and how it responds to a stress differs between tissues or organs of the same organism (Hazen et al., 2003).

### ROS-Related DEGs are Overrepresented Among the DEGs Detected by MA.1 and MA.2

Although the DEGs in response to different stresses varied greatly, the proportion of those that were *a priori* considered as ROS-related was rather constant (**Table 2**). And while these ROS-related genes correspond to only 4% of the rice expressed genes, this category accounted for up to 10% the shared DEGs, highlighting the importance of ROS scavenging and signaling to the response to stress (**Table 3C**). Interestingly, the proportion was similar in OS and AS experiments. ROS imbalance or oxidative bursts that characterize OS are often indirect consequences of another environmental stresses (Guo et al., 2006). In the MA.2 approach, direct OS were separated and compared with other AS, such as salinity, drought, P deficiency, or Fe toxicity (AS). Ozone and hydrogen peroxide were used to generate the OS in the OS experiments. While hydrogen peroxide is a normal product of the plant metabolism, tropospheric ozone mostly originates from anthropogenic gas emissions (Baier et al., 2005; Wang and Frei, 2011). Most of the damage caused by this air pollutant is caused by its immediate degradation into ROS in the apoplastic space of plant cells, including hydrogen peroxide. Directly and indirectly O_3_ induces a cascade of active ROS production and signaling (Vaultier and Jolivet, 2015). Both OS treatments thus induce direct OS, though from opposite starting sites. While the O_3_ treatment involved fumigating of leaves (Frei et al., 2010a; Cho et al., 2013), the H_2_O_2_ treatment was performed by growing seedlings in a solution containing 10 mM H_2_O_2_, starting the oxidative reactions from the plant roots (Mittal et al., 2012a,b).

Despite the theoretical differences between OS and AS experiments, the percentage of DEGs considered to be ROS-related was similar (**Tables 2** and **3**). This data enforces the concept that most AS generate OS or ROS signaling to some degree and highlights the importance of ROS homeostasis in AS response. Diverse studies claimed associations of OS with the stresses included in this MA: drought (Noctor et al., 2014); salinity (Miller et al., 2010; Chawla et al., 2012); temperature extremes (Guo et al., 2006; Mittal et al., 2012a); zinc deficiency (Frei et al., 2010b; Höller et al., 2014); phosphorous deficiency (Hernandez and Munne-Bosch, 2015); potassium deficiency (Cakmak, 2005; Ma et al., 2012), and iron deficiency (Zheng et al., 2009); iron toxicity (Matthus et al., 2015); cadmium toxicity (Uraguchi et al., 2011; Ogo et al., 2014); arsenic toxicity (Azizur Rahman et al., 2007); and lead toxicity (Li et al., 2012). In agreement with these studies, our results suggest that ROS homeostasis plays a similarly important role in all of these stresses.

### Putative Functions of Multi-Stress-Responsive Genes Detected by Combined MA Approaches

Differentially expressed genes detected by both approaches (i.e., MA.1 and MA.2) shared many GO terms, and even if the individual DEGs detected by each approach diverged (**Figures 1** and **4**), their functions were rather conserved. The DEGs identified by MA.1 and MA.2 were mostly related to metabolic and cellular processes, but also involved in the response to stimuli, regulation, transcription, and transport processes. Many of those genes can be associated with signaling pathways possessing functions such as catalytic, transferase, hydrolase, transport activity, DNA binding, and transcript regulator activity (**Figure 3**). Interestingly, the terms signal transduction process, metal ion binding activity, transcription factor activity and pyrophosphatase activity were found only among OS DEGs (**Figure 4**).

As the GO enrichment analysis demonstrated, the DEGs elected by these MA approaches represented different classes of proteins and are involved in many distinct processes. In MA.2, 311 genes were identified in both OS and AS. Between those genes, a great number of distinct transporters were present, such as the calcium transporter ATPases LOC_Os12g39660 and LOC_Os05g02940, the citrate transporter protein LOC_Os02g57620, the inorganic phosphate transporter LOC_Os02g38020, the sulfate transporter LOC_Os03g09930, the amino acid transporter LOC_Os06g36180, the aquaporin LOC_Os02g41860, and others (Supplementary Table S5). Among those were also four HIPPs, proteins that can act as cytoplasmic transporters of metallic ions and co-factor in the transcription of many stress related genes (de Abreu-Neto et al., 2013).

Using the network tool STRING 10.0, we identified a cluster of 36 DEGs that are mostly involved in the photosynthetic apparatus and its regulation (**Figure 2**). Photosynthesis is a main source of ROS in plants, which can be enhanced by AS, leading to oxidative damage if not controlled (Foyer and Shigeoka, 2011; Voss et al., 2013). Interestingly, only one of those DEGs is directly involved in the ROS scavenging pathway. The cytosolic monodehydroascorbate reductase 2 (MDAR2), coded by LOC_Os08g44340, is an enzyme that regenerates ascorbate back from its oxidized form (monodehydroascorbate; Noctor et al., 2014). Most genes identified by this “interactomic approach” did not belong to this group of well-studied antioxidants and antioxidant enzymes, e.g., AsA, GSH, Trx, CAT, APX, and GPX (Foyer and Shigeoka, 2011; Maruta et al., 2012). Instead, the elected genes were directly involved in the photosynthetic apparatus or regulatory elements, such as WRKY, MYB, and TYFF transcription factors (Supplementary Table S5). More than half of these genes showed a similar expression pattern, being highly expressed in response to iron deficiency and drought in roots, while suppressed in As toxicity, ozone, and submergence (**Figure 3**). Curiously, they were induced in roots and shoots under drought stress, but repressed in seedlings in the same treatments. This apparent contradiction could reflect differences in the age of the samples or the methods used to simulate the stress. In the experiments where roots and shoots were collected, the hydroponic solution in which the plants grew were slowly drained (Wang et al., 2011), while the whole seedlings were dried in tissue paper (Jain et al., 2007). *MDAR2* did not represents the same expression pattern as this group of photosynthesis-related genes (**Figure 3**) and was positioned as a terminal node of the predicted cluster, connected only with LOC_Os06g0539, that codes a plastid gene of unknown function (**Figure 2B**). Although we cannot be certain about the involvement of *MDAR2* with the others DEGs of this cluster, many studies have shown the importance of MDAR enzymes to the response and tolerance to AS (Sultana et al., 2012; El Airaj et al., 2013).

Among the DEGs shared between OS and AS, which did not fall into that cluster, other genes possessing hub roles (connecting distinct signaling and metabolic pathways) were also identified, for example OsSRO1c (Similar to Radical-Induced Cell Death-One 1c, LOC_Os03g12820). Radical-induced Cell Death1 (*AtRCD1*) received its name due to the ozone hypersensitive phenotype observed in plant knock-outs to this gene (Ahlfors et al., 2004; Miao et al., 2006). AtRCD1 activity is modulated through oxidation by a GPX (AtGPX3; Ahlfors et al., 2004; Miao et al., 2006). Recent studies have demonstrated that GPX proteins play important roles as redox sensors and connect ROS signaling with hormonal signaling pathways (Fourquet et al., 2008; Passaia et al., 2013; Passaia and Margis-pinheiro, 2015). One way this connection occurs is by SRO proteins (SRO), that were shown to interact with many different transcription factors (e.g., DREB2A and COL10) and are involved in transcription factor regulation and complex formation (Ahlfors et al., 2004; Jaspers et al., 2009). AtRCD1 plays a role in the plant development and response to stress, mutants experiments show the participation of this protein in ethylene, ROS, salicylic acid, abiscisic acid (ABA), and jasmonic acid (JA) signaling pathway (Ahlfors et al., 2004; Jaspers et al., 2009, 2010). A recent study have demonstrated that OsSRO1c is induced in response to multiple stresses and was show to improve drought and OS tolerance by promoting stomatal closure and H_2_O_2_ accumulation (You et al., 2013).

## Conclusion

A MA approach integrating different statistical methods allowed us to narrow down shared DEGs to a relatively small number that should be further investigated in detail. The comparison of shared DEGs with a list of genes *a priori* considered to be ROS-related highlighted the importance of redox homeostasis in stress response and signaling. Among the shared DEGs identified in this study are interesting candidates such as OsSRO1c, which regulate a great number of other proteins and connect different signaling pathways.

## Conflict of Interest Statement

The authors declare that the research was conducted in the absence of any commercial or financial relationships that could be construed as a potential conflict of interest.

The reviewer Jun You and handling Editor Zhulong Chan declared their shared affiliation, and the handling Editor states that, nevertheless, the process met the standards of a fair and objective review.

## Footnotes

1 http://www.ricearray.org/

2 http://www.ncbi.nlm.nih.gov/geo/
